# Case report: high-dose carvedilol as a potential key drug for arrhythmias in histiocytoid cardiomyopathy

**DOI:** 10.1093/ehjcr/ytad588

**Published:** 2023-11-27

**Authors:** Rei Yoshida, Heima Sakaguchi, Yoshiaki Kato, Kenichi Kurosaki

**Affiliations:** Department of Pediatric Cardiology, National Cerebral and Cardiovascular Center Hospital, 6-1 Kishibeshimmachi, Suita, 5648565 Osaka, Japan; Department of Pediatric Cardiology, National Cerebral and Cardiovascular Center Hospital, 6-1 Kishibeshimmachi, Suita, 5648565 Osaka, Japan; Department of Pediatric Cardiology, National Cerebral and Cardiovascular Center Hospital, 6-1 Kishibeshimmachi, Suita, 5648565 Osaka, Japan; Department of Pediatric Cardiology, National Cerebral and Cardiovascular Center Hospital, 6-1 Kishibeshimmachi, Suita, 5648565 Osaka, Japan

**Keywords:** Case report, Histiocytoid cardiomyopathy, Refractory ventricular tachycardia, High-dose carvedilol

## Abstract

**Background:**

Histiocytoid cardiomyopathy is a rare infancy cardiac disorder manifesting as severe cardiac arrhythmias or dilated cardiomyopathy. There is no specific treatment for these arrhythmias. This is the first report of infantile histiocytoid cardiomyopathy whose refractory ventricular arrhythmias were successfully controlled by high-dose carvedilol.

**Case summary:**

A 4-month-old girl presented with asystole, and recurrent ventricular tachycardias. From the histological findings and clinical symptoms, she was diagnosed as histiocytoid cardiomyopathy. Sedatives were the most effective therapy for her arrhythmia, but the cardiac sympathetic denervation was not effective enough. Finally, her ventricular arrhythmias were controlled with high-dose carvedilol, and she was discharged on hospitalization Day 393.

**Discussion:**

Carvedilol is the only beta blocker that directly acts on the ryanodine receptor (RyR2) and inhibits store-overload-induced Ca^2+^ release (SOICR) in myocardium at high dosage. The arrhythmias did not disappear with bisoprolol, landiolol, or verapamil, but high-dose carvedilol was effective. This clinical course suggested that the arrhythmias in histiocytoid cardiomyopathy might be related with SOICR. High-dose carvedilol might be a key drug for patients with histiocytoid cardiomyopathy.

**Learning points**

- The arrhythmia control in histiocytoid cardiomyopathy is challenging with no specific therapy.
- High-dose carvedilol that directly inhibits store-overload-induced Ca^2+^ release might be a key drug for patients with histiocytoid cardiomyopathy.

## Introduction

Histiocytoid cardiomyopathy, first described by Voth^1^, is a rare, genetic infancy or childhood cardiac disorder that mainly affect females younger than 2 years, and manifest as severe cardiac arrhythmias or dilated cardiomyopathy.^2–4^ Autosomal recessive, X-linked, and maternal inheritance has been described in the previous reports.^3^ Extracardiac manifestations include abnormalities of the central nervous system, the eyes, the endocrine systems, or steatosis of the liver.^3^ The diagnosis is established based on these clinical findings and myocardial biopsy or autopsy.^3^ There is no specific treatment for arrhythmias of histiocytoid cardiomyopathy. Cataldo *et al.*^5^ and Hirano *et al.*^6^ reported the cases of histiocytoid cardiomyopathy treated with radiofrequency ablation but their outcomes were not satisfactory.

## Summary figure

**Figure:**
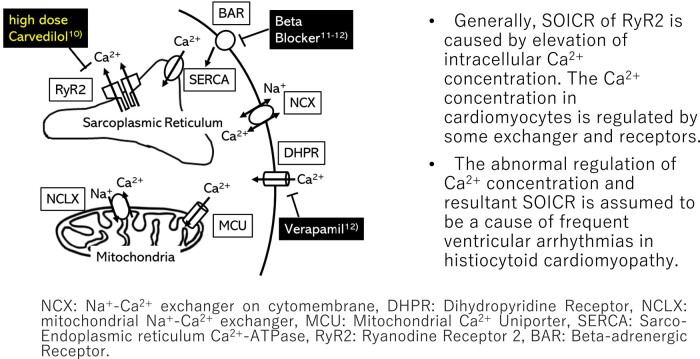


We report a case of histiocytoid cardiomyopathy with refractory ventricular tachycardia that was successfully controlled by high-dose carvedilol.

## Case presentation

A 4-month-old Asian girl was transferred to the previous hospital due to fever and episodic seizure after vaccination. She was asystole on arrival, and after intravenous administration of adrenaline and cardiac resuscitation, she had ventricular fibrillation requiring several defibrillations. She was transferred to our hospital on suspicion of myocarditis. She had no family histories of arrhythmias, cardiomyopathies, or sudden cardiac death.

**Figure 1:**
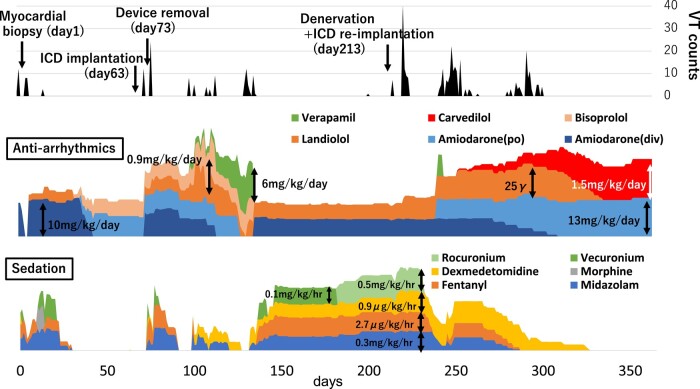
Clinical course. Horizontal axis: days after admission to our hospital. Upper row: counts of ventricular tachycardia. Middle row: doses of anti-arrhythmic drugs. Lower row: doses of sedatives. VT, ventricular tachycardia; po, per os; div, dropped intravenously.

**Figure 2:**
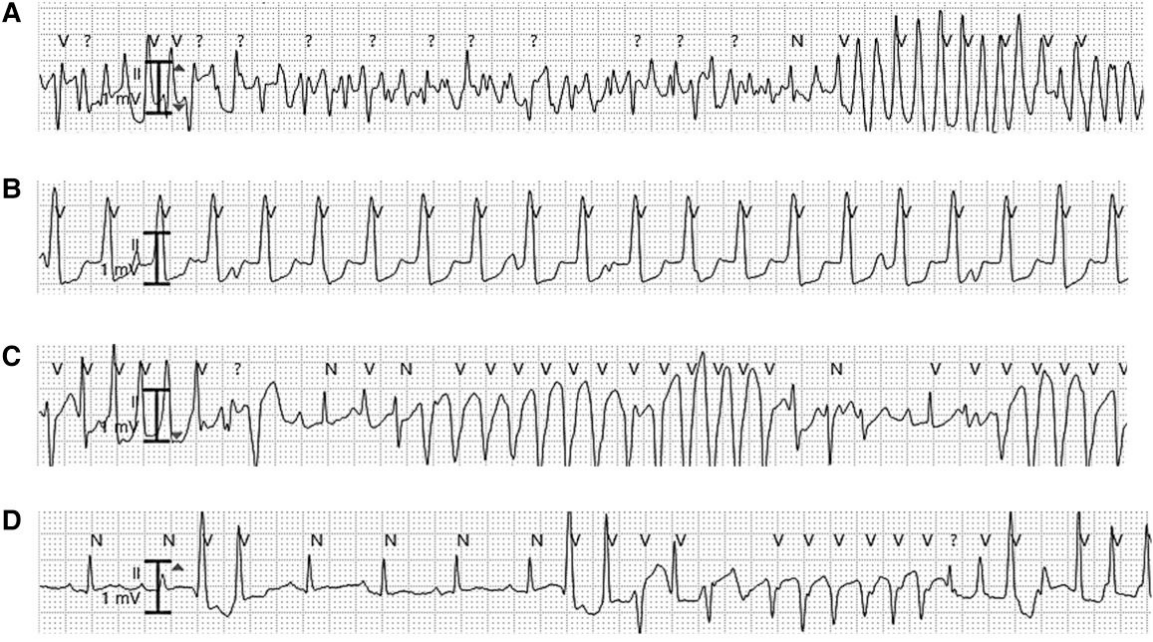
Monitored ventricular tachycardias. (*A*) Ventricular fibrillation, (*B*) slow ventricular tachycardia, (*C*) polymorphic ventricular tachycardia, (*D*) short-coupled variant of torsade de pointes.

**Figure 3:**
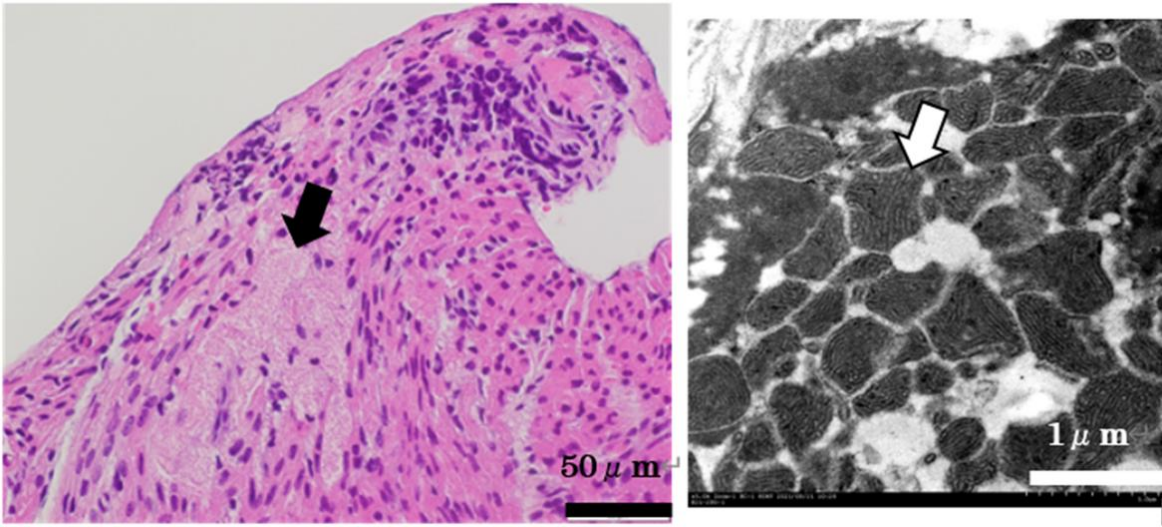
Left: haematoxylin–eosin stain. The arrow indicates histiocytoid cells. Right: electron microscopic findings. The arrow indicates abnormal mitochondria.

The clinical course is shown in *Figure 1*. Her heart rate was elevated to 140 beats per min (sinus rhythm), blood pressure was 84/56 mmHg, and percutaneous oxygen saturation (SpO_2_) was 96%. On physical examination, she had no external malformations, inspiratory retraction, nor heart murmur. The echocardiography showed no anomalies of the heart nor the great vessels. Her blood test showed no abnormalities of electrolytes including potassium, calcium, and magnesium. C-reactive protein was elevated to 7.03 mg/dL, but leucocyte count was normal and there was no elevation of creatinine kinase. For definitive diagnosis, myocardial biopsy was performed on Day 1. During biopsy ventricular tachycardia (VT) was easily induced by catheter manipulation, and intermittent sustained or non-sustained VT was observed in the critical care unit (*Figure 2A*); therefore, continuous intravenous infusion of amiodarone, landiolol, and deep sedation was started. Histological work-up using a light microscope showed large, round polygonal cells (histiocytoid cells) clustered under the endocardium, and the examination with electron microscope revealed numerous enlarged mitochondria in the histiocytoid cells (*Figure 3*). From these findings and clinical course, she was diagnosed as histiocytoid cardiomyopathy. Since VT disappeared on Day 15 with oral amiodarone and bisoprolol, an implantable cardioverter defibrillator (ICD) was implanted for secondary prevention on Day 63.

On Day 73, however, the ICD systems had to be removed due to infection of the device pocket. Frequent VT or fibrillation recurred during and after the operation, and intravenous amiodarone, landiolol, and sedatives were administered again. A month later with tapering the sedatives, various types of VT manifested, including junctional tachycardia, torsade de pointes, and short-coupled variant of torsade de pointes (*Figure 2B*–*D*) therefore, oral verapamil was added on, and amiodarone was weaned off. On Day 132 with tapering the sedatives, however, VT recurred and required sedation and intravenous amiodarone again. Oral verapamil and bisoprolol were thought to be ineffective and were stopped. Under deep sedation, the arrhythmias were completely controlled with intravenous amiodarone and landiolol. Since sedatives appeared to be more effective than anti-arrhythmic drugs for her arrhythmias, cardiac sympathetic denervation therapy^7,8^ was scheduled.

Due to a lack of experience with ganglion blockade^7^ and sympathetic denervation^8^ for the refractory ventricular arrhythmia of children (including the other hospitals in our country), the aorta and pulmonary artery were divided and each anastomosed,^9^ and the ICD system was re-implanted on Day 213 after approval from the hospital ethics committee. She developed frequent VT for 2 weeks after the surgery because of systemic or local inflammation. Intravenous magnesium sulfate and potassium chloride were partially effective but could not control her arrhythmias completely.

From Day 253, oral carvedilol 0.05 mg/kg/day, an inhibitor of store-overload-induced Ca^2+^ release (SOICR) in the myocardium,^10^ was started and was increased gradually, while the sedatives were concurrently tapered. When carvedilol was increased to more than 0.8 mg/kg/day, VT decreased significantly, and no PVCs were seen at the dose of 1.5 mg/kg/day. Finally, oral carvedilol 1.5 mg/kg/day and amiodarone 13 mg/kg/day completely controlled her VT without ventricular dysfunction and bradycardia (with a help of AAI pacing).

Other than arrhythmias, she suffered from severe hypoxic encephalopathy, epilepsy, and liver steatosis. After epilepsy was controlled with phenobarbital and levetiracetam, she was discharged on Day 393. She readmitted because of pneumonia several times but VT has not been recorded since discharge.

## Discussion

Histiocytoid cardiomyopathy is a rare infancy or childhood disorder, ∼20% of which present as sudden death.^2–4^ Pathohistologically, histiocytoid cardiomyopathy is characterized by the development of abnormal Purkinje fibres, which result in subendocardial yellow-tan nodules built up of histiocytoid myocytes, which are filled with abnormal mitochondria.^3,4^ Generally, arrhythmia control in histiocytoid cardiomyopathy is challenging with no specific therapy.^5,6^ To our knowledge, this is the first case report of histiocytoid cardiomyopathy with refractory ventricular arrhythmias controlled by oral medication.

At first, cardiac denervation was thought to be effective because deep sedation was more effective than anti-arrhythmic drugs. Studies have shown that the autonomic nervous system has an important role in triggering and maintaining ventricular arrhythmia, and cardiac sympathetic intervention by stellate ganglion blockade or surgical resection of extrinsic sympathetic chain may reduce the burden of ventricular arrhythmias.^7,8^ Cataldo *et al.*^5^ reported a case of histiocytoid cardiomyopathy in which sympathetic denervation by removal of T2–T3 stellate ganglion had been performed but was ineffective. Without the experience of these techniques in our hospital, we chose open surgery in which the aorta and pulmonary artery were divided and anastomosed each (similar to heart transplant^9^). However, after denervation, VT was frequently seen with lower dose sedatives. This approach might be ineffective for this kind of arrhythmia.

The key drug of this case was carvedilol. In this case, bisoprolol, landiolol, and verapamil could not control ventricular arrhythmia. In order to control the arrhythmias with oral medication, we chose carvedilol that is the only beta blocker inhibiting store-overload-induced Ca^2+^ release (SOICR) in the myocardium.^10^ Ventricular tachycardia was observed with <0.8 mg/kg/day carvedilol, but disappeared with a higher dose, therefore high-dose carvedilol played an important role for control of her arrhythmias.

The SOICR is a cause of triggered activity, therefore, carvedilol is thought to be more effective than the other beta blockers for catecholaminergic polymorphic ventricular tachycardia (CPVT).^11^ Verapamil and other beta blockers can also suppress SOICR by acting on dihydropyridine receptor (DHPR) and beta-adrenergic receptor (BAR) each.^11,12^ In contrast, carvedilol directly acts on the cardiac ryanodine receptor (RyR2).^10^ However, the concentrations of carvedilol required to suppress SOICR are much higher than those for beta blockade, therefore, strong inhibition of SOICR requires high doses of carvedilol.^10^ The clinical course of our case suggests that the SOICR of RyR2 is one of the causes of her arrhythmias, consequently high-dose carvedilol was effective for her. RyR2 gene mutations are often reported in cases of CPVT but were negative based on her blood sample.

**Figure 4:**
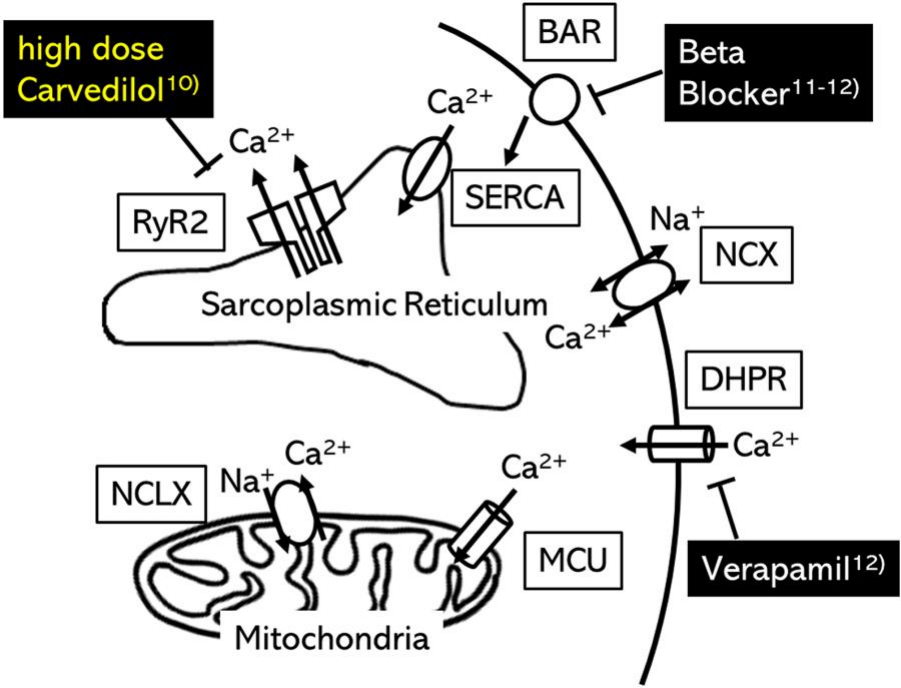
Ca^2+^ regulation in cardiomyocytes. The figure is modified from Takeuchi *et al.*^13^ NCX, Na^+^–Ca^2+^ exchanger on cytomembrane; DHPR, dihydropyridine receptor; NCLX, mitochondrial Na^+^–Ca^2+^ exchanger; MCU, mitochondrial Ca^2+^ uniporter; SERCA, sarco-endoplasmic reticulum Ca^2+^-ATPase; RyR2, ryanodine receptor 2; BAR, beta-adrenergic receptor.

Generally, SOICR of RyR2 is caused by elevation of intracellular Ca^2+^ concentration. The Ca^2+^ concentration in cardiomyocytes is regulated by some exchanger and receptors on cytomembrane, sarcoplasmic reticulum, and mitochondria (*Figure 4*).^13,14^ The abnormal mitochondria in cardiomyocytes in histiocytoid cardiomyopathy and resulting in abnormal regulation of intracellular Ca^2+^ concentration may be associated with their refractory ventricular tachycardias.

To our knowledge, there is no report of successful control of arrhythmias in histiocytoid cardiomyopathy with high-dose carvedilol. High-dose carvedilol that directly inhibits SOICR might be a key drug for patients with histiocytoid cardiomyopathy.

## Data Availability

The data underlying this article will be shared on reasonable request to the corresponding author.

## Lead author biography

Rei Yoshida graduated from Faculty of Medicine, Tokyo University, Tokyo, Japan in March 2015. Experience: 2017–2020: Department of Pediatrics, Okinawa Chubu Hospital, Okinawa, Japan. 2020–Present: Department of Pediatric Cardiology, National Cerebral and Cardiovascular Center Hospital, Osaka, Japan. Licenses: A Board Certified Pediatrician of the Japanese Society of Pediatrics, February 2022.
